# Post-procedure Rhinitis After Use of Sedatives and Supplemental Nasal Oxygen

**DOI:** 10.7759/cureus.23397

**Published:** 2022-03-22

**Authors:** Sharmela Brijmohan, Tanganyika Barnes

**Affiliations:** 1 Internal Medicine, Englewood Hospital and Medical Centre, Englewood, USA

**Keywords:** post-procedure, oxygen, esophagogastroduodenoscopy, nasal cannula, rhinitis

## Abstract

Post-procedure rhinitis is a form of nonallergic rhinitis that is a common but underreported complication occurring after procedures requiring sedation and the use of supplemental oxygen via nasal cannula. Symptoms occur immediately after awakening from sedation and may be so unbearable, that it impairs work, daily functioning, sleep patterns, and quality of life. This case describes a 67-year-old male who developed these symptoms after esophagogastroduodenoscopy (EGD) under sedation with supplemental oxygen via nasal cannula. The proposed pathophysiology is thought to be due to the impingement of the cannula against mechanoreceptors in the nasal mucosal membrane causing parasympathetic overactivity. Based on this pathogenesis, the reported patient failed to show improvement with oral antihistamines but instead benefited from a topical anticholinergic agent. Patients at risk should be informed of the possibility of this adverse event and advised on proper treatment if it occurs.

## Introduction

Many procedures are performed with some form of sedation. However, opioids, benzodiazepines, and short-acting anesthetics such as propofol can lead to hypoventilation. The possibility of hypoxia may be prevented by administering supplemental oxygen via various delivery devices including nasal cannula [1]. This article describes an underreported complication of nonallergic rhinitis after sedation with propofol and the use of supplemental oxygen via nasal cannula. An article published by Li et al. revealed symptoms in 7.1% of patients [2]. Our literature review revealed two published case reports and one clinical trial addressing this issue [2-4]. According to Cohen et al., the incidence of post-procedural rhinitis may be higher than the number of publications on the subject [4].

Over 183 patients as of August 01, 2021, can also be found reporting similar symptoms on a Google blog post entitled “Sneezing, runny nose and tearing after colonoscopy” [5]. These patients reported abrupt onset of unbearable symptoms immediately after awakening from deep sedation. The patient reported that their symptoms caused significant disruption to everyday life and felt that caregivers underestimated their symptoms. We believe this is a common adverse event that is underreported because caregivers are not familiar with this problem. Annual healthcare expenses related to rhinitis treatment are estimated to be >$4.6 billion [6]. Our goal is to increase awareness among patients and caregivers; thus improving patient satisfaction rates, decreasing the economic burden, and decreasing the negative impact on the patients’ quality of life (QOL) [7-10].

## Case presentation

**Figure 1:**
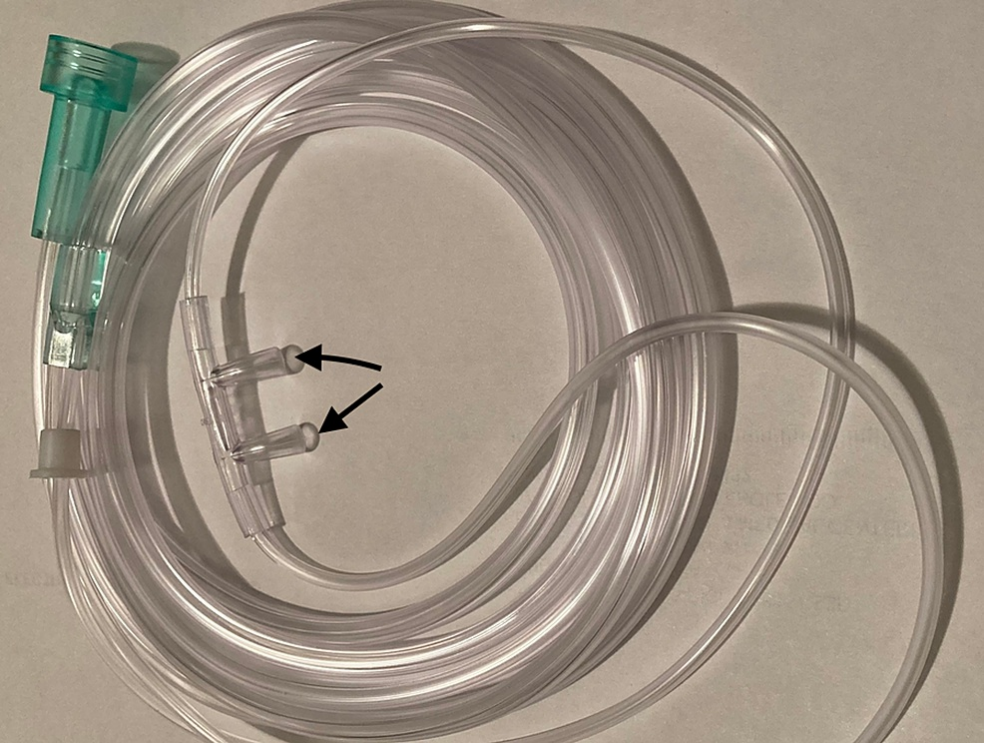
Nasal cannula that was used for supplemental oxygen during sedation. Arrows point to 10-mm long nasal prongs.

A 67-year-old male patient with a past medical history of alcoholic cirrhosis recently discovered a 2.7 cm mass in the tail of the pancreas, chronic obstructive pulmonary disease (COPD), and seasonal allergies was admitted for *Clostridioides difficile* colitis after presenting with persistent diarrhea and abdominal pain. His treatment regimen included oral vancomycin for colitis; fluticasone-furoate-vilanterol, tiotropium, and albuterol inhalers for his COPD; fluticasone nasal spray and loratadine for seasonal allergies. On day three of hospitalization, an esophagogastroduodenoscopy (EGD) with endoscopic ultrasound-guided biopsy was performed due to concerns for underlying esophageal varices and pancreatic malignancy. The patient underwent rapid sequence intubation and was maintained on total intravenous anesthesia (TIVA) with propofol and dexmedetomidine. Post-extubation, oxygen saturation was maintained with supplemental oxygen via a silicone-based nasal cannula with 10 mm prongs at a flow rate of 4 L/min (Figure 1).

On awakening from the procedure, the patient complained of severe bouts of sneezing, unilateral clear, watery nasal discharge, and lacrimation. In less than 12 hours, he used two tissue boxes of 100 sheets each, with little relief of symptoms from fluticasone nasal spray, loratadine, fexofenadine, and diphenhydramine. Due to persistent symptoms and poor sleep, he refused to be discharged and requested treatment with antibiotics. He was started on intranasal ipratropium bromide (0.03% solution), two sprays in each nostril three times daily which improved his symptoms. In total, he spent an additional three days in the hospital. The EGD showed no varices but biopsy of the pancreatic mass showed poorly differentiated ductal adenocarcinoma.

## Discussion

Rhinitis is an inflammation of the nasal mucous membrane. It is often categorized as allergic, infectious, or nonallergic. Symptoms include nasal congestion, sneezing, rhinorrhea, and postnasal drainage [11]. Some view rhinitis as a trivial disease, but it may impair work, daily functioning, sleep patterns, and QOL [9-10]. Additionally, it can cause socioeconomic burdens due to medications cost and indirect costs related to absenteeism and presentism at work [7-8].

Many patients have reported symptoms of rhinitis upon waking up from endoscopy requiring sedation and supplemental oxygen via nasal cannula [5]. Cohen et al. reported similar symptoms in a 66-year-old woman upon awakening from sedation and supplemental nasal oxygen for cataract extraction. They referred to this phenomenon as “PRAISE SNOG,” which stands for “Post-procedural Rhinitis After Intravenous Sedation With Supplemental Nasal Oxygen” [4]. A literature review suggests the pathophysiology may be due to nonallergic rhinitis [4]. Nonallergic rhinitis, unlike allergic rhinitis, is not immunoglobulin E-mediated, but rather the symptoms are triggered by a non-immunologic stimulus causing mast cell activation and autonomic stimulation [2]. This non-immunologic stimulus is thought to be due to the oxygen nasal prongs causing mucosal injury when it impinges against the membrane in a sedated patient who cannot intervene to readjust the prongs [3]. Mucosal injury leads to stimulation of nasal mechanoreceptors and/or C-fibers, leading to parasympathetic overactivity, resulting in rhinitis symptoms. This is further aggravated by the drying and cold oxygen flow on the nasal mucous [3].

The diagnosis is one of exclusion. In the studies reviewed, some patients reported a medical history of rhinitis while others did not, suggesting a nonallergic nature of their symptoms [2-4]. The common theme is the acute onset of rhinitis symptoms post-procedure with sedation and supplemental oxygen via a nasal cannula, that lasts for days to months. One may question whether these symptoms could be explained by cold air rhinitis from the temperature of the examination room or the oxygen itself. Li et al. investigated this possibility in a clinical trial involving 836 patients, some of whom developed rhinitis symptoms after sedation for endoscopy, and found that the temperature of the room and the oxygen was about 24-25°C; which is far warmer than the air that induces cold air rhinitis [2].

In the case above, the patient reported little relief of symptoms from fluticasone nasal spray, loratadine, fexofenadine, and diphenhydramine. A review of the treatments tried by patients of the Google blog post entitled “Sneezing, runny nose and tearing after colonoscopy” revealed that many patients also claimed little relief from antihistamines such as diphenhydramine, loratadine, and cetirizine [5]. Studies have shown that second-generation oral antihistamines are not as effective in treating nonallergic rhinitis. However, first-generation oral antihistamines may have some benefits due to anticholinergic activity [11-12]. Based on the proposed pathophysiology, Rah and Merkel suggested that topical anticholinergic spray may be more effective than antihistamine [3]. Cohen et al. and Li et al. recommended using trimmed nasal cannula, awake sedation, nasal or face mask where possible [2,4]. Further studies are needed to determine if these patients could benefit from novel techniques such as allergen immunotherapy (AIT), posterior nasal nerve (PNN) cryoablation, and electroacupuncture [13-15].

## Conclusions

Impingement of nasal oxygen cannula against the nasal mucosal membrane in a sedated patient can result in nonallergic rhinitis. Based on this pathogenesis, topical anticholinergic agents may be effective. We believe at-risk patients should be informed of the possibility of this adverse event, advised on proper treatment, and have it documented in their chart when it occurs. Further studies are needed to evaluate effective treatment options and prevention.

## References

[1] Lin OS (2017). Sedation for routine gastrointestinal endoscopic procedures: a review on efficacy, safety, efficiency, cost and satisfaction. Intest Res.

[2] Li NL, Tseng SC, Hsu CC (2011). A simple, innovative way to reduce rhinitis symptoms after sedation during endoscopy. Can J Gastroenterol.

[3] Rah KH, Merkel IS (2019). Unilateral rhinorrhea and sneezing after upper gastrointestinal endoscopy under intravenous propofol sedation with supplemental oxygen administered via a nasal cannula: a case report. A A Pract.

[4] Cohen PR, Coden DJ, Kurzrock R (2021). Bilateral postprocedural rhinitis after intravenous sedation with supplemental nasal oxygen (PRAISE SNOG) after cataract surgery. Cureus.

[5] (2021). Sneezing, runny nose and tearing after colonoscopy. Dec.

[6] Roland LT, Wise SK, Wang H, Zhang P, Mehta C, Levy JM (2021). The cost of rhinitis in the United States: a national insurance claims analysis. Int Forum Allergy Rhinol.

[7] Lamb CE, Ratner PH, Johnson CE (2006). Economic impact of workplace productivity losses due to allergic rhinitis compared with select medical conditions in the United States from an employer perspective. Curr Med Res Opin.

[8] Dierick BJ, van der Molen T, Flokstra-de Blok BM, Muraro A, Postma MJ, Kocks JW, van Boven JF (2020). Burden and socioeconomics of asthma, allergic rhinitis, atopic dermatitis and food allergy. Expert Rev Pharmacoecon Outcomes Res.

[9] Vandenplas O, Suarthana E, Rifflart C, Lemière C, Le Moual N, Bousquet J (2020). The impact of work-related rhinitis on quality of life and work productivity: a general workforce-based survey. J Allergy Clin Immunol Pract.

[10] Cuesta-Herranz J, Laguna JJ, Mielgo R (2019). Quality of life improvement with allergen immunotherapy treatment in patients with rhinoconjunctivitis in real life conditions. Results of an observational prospective study (ÍCARA). Eur Ann Allergy Clin Immunol.

[11] Beard S (2014). Rhinitis. Prim Care.

[12] Tran NP, Vickery J, Blaiss MS (2011). Management of rhinitis: allergic and non-allergic. Allergy Asthma Immunol Res.

[13] Steele TO, Hoshal SG, Kim M (2020). A preliminary report on the effect of gabapentin pretreatment on periprocedural pain during in-office posterior nasal nerve cryoablation. Int Forum Allergy Rhinol.

[14] Gerka Stuyt JA, Luk L, Keschner D, Garg R (2021). Evaluation of in-office cryoablation of posterior nasal nerves for the treatment of rhinitis. Allergy Rhinol.

[15] Fu L, Zhong J, Fu Q, Yang Y, Zhang M, Zhang Q (2020). Clinical effects and safety of electroacupuncture for the treatment of allergic rhinitis: a protocol for systematic review. Medicine.

